# Predicting Outcomes in Emergency Medical Admissions Using a Laboratory Only Nomogram

**DOI:** 10.1155/2017/5267864

**Published:** 2017-11-14

**Authors:** Seán Cournane, Richard Conway, Declan Byrne, Deirdre O'Riordan, Bernard Silke

**Affiliations:** ^1^Medical Physics and Bioengineering Department, St. James's Hospital, Dublin 8, Ireland; ^2^Department of Internal Medicine, St. James's Hospital, Dublin 8, Ireland

## Abstract

**Background:**

We describe a nomogram to explain an Acute Illness Severity model, derived from emergency room triage and admission laboratory data, to predict 30-day in-hospital survival following an emergency medical admission.

**Methods:**

For emergency medical admissions (96,305 episodes in 50,612 patients) between 2002 and 2016, the relationship between 30-day in-hospital mortality and admission laboratory data was determined using logistic regression. The previously validated Acute Illness Severity model was then transposed to a Kattan-style nomogram with a Stata user-written program.

**Results:**

The Acute Illness Severity was based on the admission Manchester triage category and biochemical laboratory score; these latter were based on the serum albumin, sodium, potassium, urea, red cell distribution width, and troponin status. The laboratory admission data was predictive with an AUROC of 0.85 (95% CI: 0.85, 0.86). The sensitivity was 94.4%, with a specificity of 62.7%. The positive predictive value was 21.2%, with a negative predictive value of 99.1%. For the Kattan-style nomogram, the regression coefficients are converted to a 100-point scale with the predictor parameters mapped to a probability axis. The nomogram would be an easy-to-use tool at the bedside and for educational purposes, illustrating the relative importance of the contribution of each predictor to the overall score.

**Conclusion:**

A nomogram to illustrate and explain the prognostic factors underlying an Acute Illness Severity Score system is described.

## 1. Introduction

Acute Medicine involves the immediate and early specialist management of adult patients who require urgent care for medical conditions [1]. It has been acknowledged that standardizing aspects of medical management for many conditions, such as stroke and myocardial infarction, has led to improved outcomes [2, 3]. Reforms to care delivery via the establishment of acute medical admissions units (AMAU) [4–6], in addition to implementation of other structural changes [7, 8] and the presence of senior consultant interventions [9], have led to improved patient outcomes. The ability to predict in-hospital mortality from parameters gathered at time of admission has offered a mechanism for focussing limited resources on higher risk patients [10].

A difficulty with utilising predictive methods and applying decision analysis at the bedside is that it generally requires computer software for the calculations, which may render the method impractical [11]. Alternatively, mortality risk predictions, incorporating the contribution of physiological or biochemical parameter combinations, may be calculated using a simple graphic calculation tool called a nomogram [12]. Such tools have been used to predict in-hospital mortality from acute poisoning in adults in the emergency department [13], for risk stratification in Acute Coronary syndrome [14, 15] and risk evaluation for Intensive Care patients [12]. Nomograms can embody predictions derived from regression modelling; subsequent to their design, the nomogram can be printed and employed as an educational tool or for assisting personalized clinical decisions without any requirement for a bedside computer [11]. In this work we present a nomogram for predicting 30-day in-hospital mortality for all medical admission patients admitted via the emergency department of our centre. The nomogram is based on data that would be immediately available at the time of emergency medical admission with this model and then compared with a full model which includes all predictors available retrospectively.

## 2. Methods

### 2.1. Background

St James's Hospital, Dublin, serves as a secondary care centre for emergency admissions in a catchment area with a population of 270,000 adults. All emergency medical admissions were admitted from the ED to an Acute Medical Admission Unit, the operation and outcome of which have been described elsewhere [5, 16].

### 2.2. Data Collection

An anonymous patient database was employed, collating core information of clinical episodes from the Patient Administration System (PAS), the national hospital in-patient enquiry (HIPE) scheme, the patient electronic record, the emergency room, and laboratory systems. HIPE is a national database of coded discharge summaries from acute public hospitals in Ireland [17, 18]. International Classification of Diseases, Ninth Revision, Clinical Modification (ICD-9-CM), has been used for both diagnosis and procedure coding from 1990 to 2005 with ICD-10-CM used since then. Data included parameters such as the unique hospital number, admitting consultant, date of birth, gender, area of residence, principal and up to nine additional secondary diagnoses, principal and up to nine additional secondary procedures, and admission and discharge dates. Additional information cross-linked and automatically uploaded to the database includes physiological, haematological, and biochemical parameters.

### 2.3. Risk Predictors

Derangement of biochemical parameters may be utilised to predict clinical outcome. We have previously derived and applied an Acute Illness Severity Score [19, 20], predicting in-hospital mortality from the following parameters recorded in the ED [21]. A weighted age adjusted score was derived; six risk groups (I–VI) were identified with cutoffs for 30-day in-hospital mortality at 1, 2, 4, 8, and 16%. We adjusted for Comorbidity using the Charlson Index [22] and disabling disease [23] IDC9/ICD10 discharge codes of 1, 2, 3, or 4 separate systems (e.g., cardiovascular, respiratory, diabetes, and renal). In addition, sepsis categories of (1) No Culture requested (2) Culture Negative, and (3) Culture Positive were examined. Triage categories, based on the Manchester Triage System [24] were Category 1 (resuscitation), Category 2 (very urgent), Category 3 (urgent), Category 4 (standard), and Category 5 (nonemergency).

In this study we have examined two sets of parameters. One parameter set, used in the “full” model, included all parameters available, including those available retrospectively, that were predictive of a 30-day in-hospital mortality. The second predictor set, used for the admission model, consisted of those predictors which would be immediately available at the time of emergency medical admission. These included age, admission triage, red blood cell distribution width (RDW), sodium, urea, and albumin.

### 2.4. Statistical Methods

Descriptive statistics were calculated for background demographic data, including means/standard deviations (SD), medians/interquartile ranges (IQR), or percentages. Comparisons between categorical variables and mortality were made using chi-square tests.

We assessed the prediction of outcomes and defined predictor variables that included age, Acute Illness Severity Score [19, 20], Charlson Comorbidity index [22], Chronic Disabling Score [23], Sepsis Status [25], and the admission triage category [24] in the full model. The admission model included the following predictors: age, admission triage, red blood cell distribution width (RDW), sodium, urea, and albumin. We employed a logistic model with robust estimate to allow for clustering; the correlation matrix thereby reflected the average dependence among the specified correlated observations [19]. Logistic regression analysis identified potential mortality predictors and then tested those that proved to be significant univariate predictors (*p* < 0.01 by Wald test). From these the multivariable fractional polynomial (MFP) logistic regression model that accurately predicted the probability of 30-day in-hospital mortality was derived. The Hanley and McNeil method was used to estimate AUROC statistics [26], and compared the area under the receiver operator curves as previously described [27]. The Kattan-style nomogram was implemented with a Stata user-written program “normolog” for binary logistic regression predictive models [28] and represented the admission model.

Adjusted odds ratios (OR) and 95% confidence intervals (CI) or IRRs were calculated for those predictors that significantly entered the model (*p* < 0.10). Statistical significance at *p* < 0.05 was assumed throughout. Stata v.13.1 (Stata Corporation, College Station, Texas) statistical software was used for analysis.

## 3. Results

### 3.1. Patient Demographics

A total of 96,305 episodes in 50,612 unique patients were admitted as medical emergencies from the hospital catchment area over the 15-year study period (2002–2016). These episodes represented all emergency medical admissions, including patients admitted directly into the Intensive Care Unit or High Dependency Unit, respectively. The proportion of males was 48.7%. The median (IQR) length of stay (LOS) was 5.2 (2.0, 13.1) days. The median (IQR) age was 62.1 (40.3, 78.4) yrs, with the upper 10% boundary at 86.1 yrs.

### 3.2. Risk Predictors Including Acute Illness Severity (Table 1, Figure 1)

Table 1 presents data from the full 30-day in-hospital mortality model, as previously described [19]. Based on the 30-day mortality outcomes (each patient counted once only, last admission if >1 admission), six risk groups were defined with 30-day mortality rates of Group I—0.2%, Group II—0.1%, Group III—0.6%, Group IV—1.8%, Group V—4.6%, and Group VI—25.2%. Other predictor variables included the Charlson Comorbidity index [22], the Chronic Disabling Score [23] and Sepsis Status [25], and the admission triage category [24].

**Figure 1:**
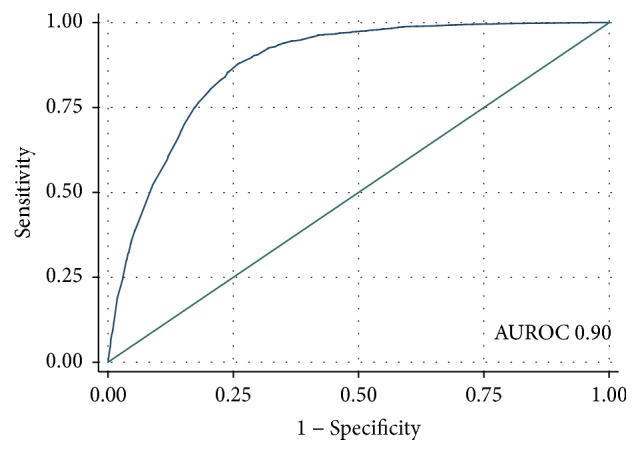
Area under the receiver operator curve (AUROC) for full model (Table 1). The sensitivity was 94.8% with a specificity of 66.8%. The positive predictive value was 23.3% with a negative predictive value of 99.2%. Patients correctly classified were 70%. The area under the receiver operator curve was 0.90 (95% CI: 0.89, 0.90).

The 30-day mortality rates for Charlson Comorbidity groups were Gr 0—2.9%, Gr I—9.4%, and Gr II—22.8%. By Chronic Disabling Score, the 30-day mortality rates were Gr 0—1.0%, Gr I—3.5%, Gr III—7.8%, Gr IV—14.3%, and Gr V—28.4%. By Sepsis Status, the 30-day mortality rates were no Blood Culture 5.5%, Blood Culture (negative) 17.7%, and Blood Culture (positive) 31.6%. By Manchester triage category, the 30-day mortality rates were Category 1 (resuscitation) 42.3%, Category 2 (very urgent) 11.3%, and Category 3 (urgent) 5.7%%. The full model was predictive with an AUROC of 0.90 (95% CI: 0.89, 0.90) (Figure 1). The sensitivity was 94.8% with a specificity of 66.8%. The positive predictive value was 23.3% with a negative predictive value of 99.2%. 70% of patients were correctly classified.

At the time of the emergency medical admission, however, the available data to the practising clinician is much more limited. The ED uses four categories of urgency with Manchester Triage Score, graded as resuscitation (2.4%), very urgent (42.5%), urgent (44.2%), and other grades (10.9%). The large majority of emergency medical admissions were classified as urgent or higher, with only 10.9% of lesser grades of urgency being admitted. The admission biochemistry and a troponin would be promptly available, but a septic screen result (if a Blood Culture is requested) would not be available for 24 hr or more. Therefore we have calculated the predictive outcome (30-day in-hospital mortality) and the nomogram on the data that is likely to be immediately available, including age, admission triage, red blood cell distribution width (RDW), sodium, urea, and albumin. The subset laboratory model was predictive with an AUROC of 0.85 (95% CI: 0.85, 0.86). The sensitivity was 94.4% with a specificity of 62.7%. The positive predictive value was 21.2% with a negative predictive value of 99.1% with 65.8% of patients correctly classified.

### 3.3. The Admission Laboratory and Triage Nomogram (Figures 2–4)

**Figure 2:**
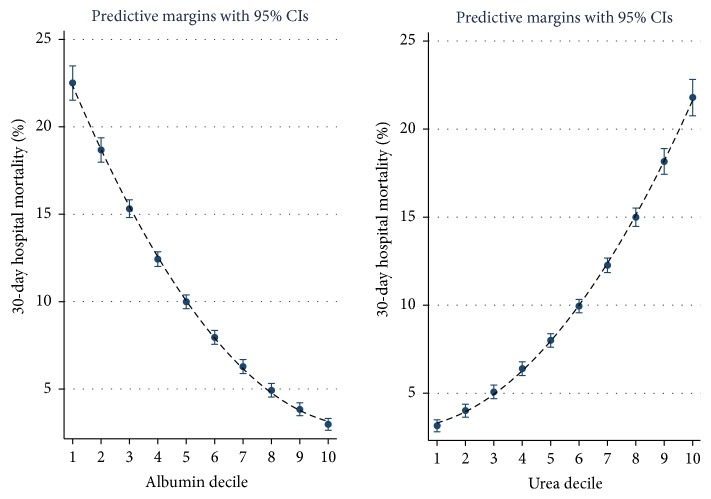
The 30-day in-hospital mortality was related to the underlying level of albumin or urea at time of admission. The decile of each predictor variable was related to the 30-day mortality rate; the risk estimate was derived from the logistic regression multivariable model and was adjusted for Charlson Comorbidity, Chronic Disabling Disease, Sepsis and Deprivation Status. We used margins to estimate the average marginal effect. The cutpoints for albumin were 31, 34, 36, 38, 39, 40, 42, 43, and 46 and for urea were 2.8, 3.6, 4.2, 4.9, 5.5, 6.3, 7.4, 9.1, and 12.7.

**Figure 3:**
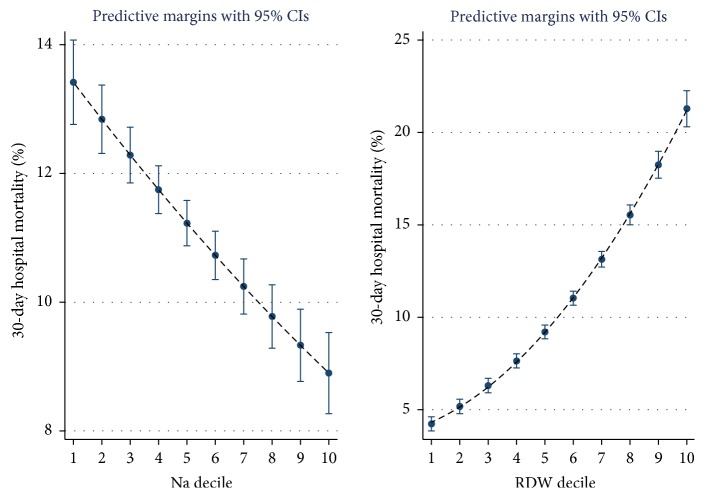
The 30-day in-hospital mortality was related to the underlying admission level of sodium or red cell distribution width (RDW). The decile of each predictor variable was related to the 30-day mortality rate; the risk estimate was derived from the logistic regression multivariable model and was adjusted for Charlson Comorbidity, Chronic Disabling Disease, Sepsis and Deprivation Status. We used margins to estimate the average marginal effect. The cutpoints for Na were 131, 134, 136, 137, 138, 139, 140, 141, and 142 and for RDW were 12.6, 12.9, 13.3, 13.6, 13.9, 14.3, 14.9, 15.7, and 17.1.

**Figure 4:**
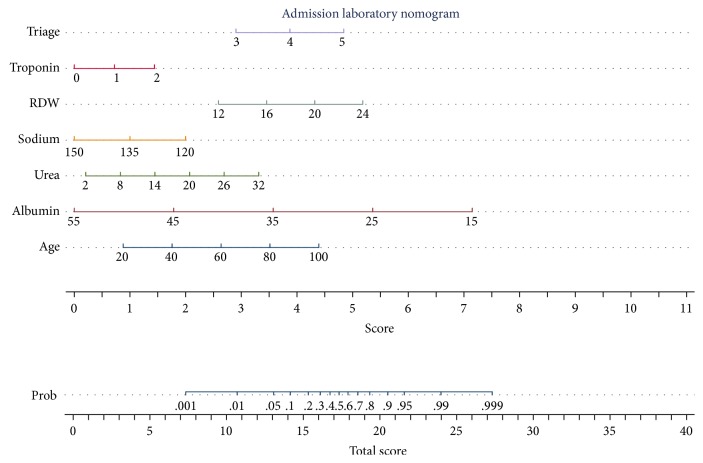
Nomogram derived from logistic regression‐based predictive models of admission triage and laboratory data. The nomogram makes output calculations from a set of input variable values much easier and the relative contribution of each to the overall score intuitively obvious. A vertical line is drawn from each input variable to the first scale (Individual Score), and the sum of these outputs is used to calculate the mortality risk from the lower scale (Total Score).

The admission laboratory data are predictive but have a different relationship to 30-day mortality outcomes. Higher albumin or sodium values predicted survival whereas higher urea or RDW deciles were associated with a worsened outcome (Figures 2 and 3). The logistic regression nomogram is derived from the logistic regression but rather than supply the full regression formula or a table with all regression coefficients, there is a visual representation. Nomograms are one of the simplest methods of mechanical calculus with a precision similar to that of a logarithmic ruler. A vertical line is drawn from each input variable to the first scale (Individual Score), and the sum of these outputs is used to calculate the mortality risk from the lower scale (Total Score) (Figure 4). For example, a patient is admitted with a triage category “urgent,” with no troponin estimation, and laboratory values of red blood cell distribution width (RDW) 18, sodium 130 mmol/l, urea 20, and albumin 30 G/dL at the age of 80 years. This would approximate to Triage 3 points, RDW 4 points, sodium 1.5 points, urea 2 points, and age 3.5 points. The total score then of 14 points would carry a 30-day mortality risk of approximately 10%, read off from the lower scale. The mortality over the last three years per patient was 7.1% (95% CI: 6.7–7.6); an 80 yr old patient, triaged as urgent, might have a 6/7 points score with the aforementioned risk; a score in the region of 15–18 points would equate to a risk of 20%.

## 4. Discussion

There has been much discussion and interest about improving the outcomes of emergency medical admissions; this may have focused on reform of Acute Medicine delivery via an acute medical admissions unit (AMAU) [4–6], or with other structural reforms [7, 8], or the presence of senior consultant interventions [9]. At our institution in 2003 we initiated reform with an AMAU with the expectation it would deliver clinical efficiency with a reduction in unit hospital length of stay without necessarily an alteration in clinical mortality outcomes. However, over the subsequent 15-yr period, there was a relative risk reduction (RRR) of 33.9% in 30-day hospital episode in-patient mortality from 7.0% to 4.6% and, when calculated on a per patient basis (count last admission if >1 episode), a there was RRR of 61.7% from 15.1% to 5.8% (between 2002 and 2016). The mortality declined essentially as a linear function over time while the hospital LOS hardly changed. This result led us to systematically examine the factors that influenced hospital mortality. Our nomogram is an attempt to clarify and simplify risk prediction and to act as an aid to understand the relative contribution of different parameters to the overall hospital mortality risk.

We have generated two different prediction models which, respectively, combine different sets of prognostic factors, including admission physiological or biochemical parameters in addition to predictor variables, such as the Charlson Comorbidity index [22], Chronic Disabling Disease Score [23], Sepsis Status [25], and the admission triage category [24]. The full model comprised all available parameters and proved predictive of a 30-day in-hospital death, returning an AUROC of 0.90 (95% CI: 0.89, 0.90). The second model, which the nomogram represents graphically, makes use of those parameters only immediately available to the practising clinician at the time of emergency medical admission, returning an AUROC of 0.85 (95% CI: 0.85, 0.86). Thus, our nomogram model offers a decision analysis tool for assisting the clinician for personalized decision-making, with very little loss in predictive accuracy as compared with the full prediction model. The illustrative nature of the nomogram allows for the intuitive understanding as to how the different predictors contribute to risk.

Laboratory data scores for predicting in-hospital mortality have previously been reported in the literature. Prytherch et al. suggested a logistic regression model based on age, mode of admission, albumin, haemoglobin, WCC, urea, electrolytes, and creatinine with their laboratory score model resulting in an AUROC of up to 0.78 and predicting death very early after admission [29]. In another single centre study, it was reported that a logistic regression model using age, albumin, alkaline phosphatase, aspartate aminotransferase, blood urea nitrogen, glucose, lactate dehydrogenase, neutrophil count, and total WCC predicted in-hospital mortality with an AUROC of 0.89 [30]. Another model combining clinical and laboratory data including age, vital signs, phosphate and albumin levels reported an AUROC of 0.84 [31]. These models were derived from studies performed and validated in single hospitals; however, some of the utilised parameters may not be routinely measured elsewhere, making their validation in different clinical centres difficult. A more widely recognised scoring system used in Acute Medical Units in the UK is the Modified Early Warning Score [19, 32]. While this score is useful for highlighting those critically ill patients who may need transfer to the Intensive Care Unit, it is more applicable to the objective of clinical deterioration prevention. Our model focuses on a general admission system which has been largely used to adjust risk between different subgroups of patients when one is looking for an intervention. Nevertheless, our model not only compares favourably with the accuracy of those models in the literature, but also presents a model combining commonly used and available data, adding to the literature in this regard.

Broadly speaking, clinical outcomes for the entire population of hospital admissions can be predicted using admission laboratory data [30, 33]. Specific important predictors may be hypo- or hypernatraemia [34–37], hypoalbuminaemia [38, 39], and hyperglycaemia [40–43]. Elevated serum urea had also been shown to be of prognostic significance [36, 44]. Our previously described Acute Illness Severity Score [19, 45] for predicting in-hospital mortality relied on admission laboratory tests (i.e., serum sodium (Na), serum potassium (K), serum urea, haematocrit or RDW, and white blood cell count (WCC)). This used a multivariate fractional polynomial method [46]. The principle is that the adjusted (for age) degree of biochemical disturbance reflects illness severity and is predictive of outcome. The admission potassium and white cell count contribute little to the overall prediction in this word leading to a reduced model being employed in the case of the nomogram.

It may be apparent that the laboratory parameters predict differently. For instance, higher albumin or sodium values predicted survival whereas higher urea or RDW deciles were associated with a worsened outcome (Figures 2 and 3). These relationships are clearly strongly predictive; however, the relationships results may be subject to bias. In the case of sodium, we have previously reported that sodium predicts mortality with a U-shaped distribution and was highest in patients whose sodium level was <125 or >140 mmol/l [34]; this U-shaped mortality curve has been well described [47]; however, this outcome is influenced by where one sets the cutoff point; in our “by decile” split of all patients, the overall relationship shows a falling overall mortality risk outcome with declining sodium. Inspecting the nomogram with this insight would explain why a patient with a sodium of 150 mmol/l is given much less weight in terms of calculating risk of 30-day mortality in comparison with the much higher weight given to a patient with an admission sodium of 120 mmol/l. It can also be appreciated that, for RDW, albumin, and urea, the smooth gradation of risk over a wider mortality risk, ensures that for these three parameter there is a wide score range evident on the nomogram, with each of these parameters contributing more to the overall prediction, compared with the sodium and the troponin assessment (troponin category—no troponin request (1), negative (2), or positive result of request (3)).

The calculation of in-hospital mortality (the proportion of those who have survived a hospital admission) can be complex. Between 2012 and 2016, for emergency medical admissions at our centre, the 30-day hospital mortality by episode was 4.6% (95% CI: 4.4–4.7); however, over this extended time period, only 34.5% of patients had a single admission; moreover approximately 5% were admitted more than 10 times. Patients admitted just once may have died, while patients admitted multiple times could only have died on their final admission. When accounting for unique patients (last admission only if >1 admission), the mortality estimate is higher at 8.9% (95% CI: 8.6–9.1). Given that the mortality rate at our institution has fallen over time, the more recent period may be more reflective of current reality. The mortality rates for the last three years, by episode or unique patients, were 4.2% (95% CI: 3.9–4.4) and 7.1% (95% CI: 6.7–7.6), respectively. Thus, the methodology for mortality rate calculation must be carefully considered when assessing the overall utility of any prediction; the predictions of this study were derived from calculations of outcome over the entire 15-year period and on a per patient basis (i.e., not on episodes). Most patients will survive the acute episode although the longer-term outcome, particularly for older persons, may be more guarded with 1-yr mortality rates approaching 20% or more [48, 49]. Though the sensitivity of the predictive algorithm for our 30-day mortality is very high, there may be many false predictions, even in high risk subsets, resulting in a much lower sensitivity.

Decision analysis can be used by clinicians to decide among alternative treatment strategies—perhaps in the acute context to stratify risk and to focus scarce resources on those at most risk [10]. As mentioned previously, applying personalized decision analysis at the bedside generally requires computer software for risk calculations and, thus, the method may prove impractical. In this work we present a nomogram which has incorporated predictions from regression modelling by combining prognostic factors to improve prediction accuracy—the nomogram can be used to apply a decision-analytic model with little loss in predictive accuracy [11].

As with any study, this work has both strengths and limitations. The strengths lie in the comprehensive nature of the data available collated over a 15-year period. Further, our aggregate score used routine laboratory tests and troponin, parameters which are often collected and available during the course of an emergency medical admission. Laboratory tests are regarded as accurate and unbiased, and are routinely available within a short period following admission. While it could be argued that different laboratories may use different methodologies and have different normal ranges, these are unlikely to be significantly different for many routine determinations, such as the full blood count and urea and electrolyte determinations. Once the data is available to the clinician, an algorithm that estimates the risk of death by day 30 can divide patients into risk groups. We have suggested setting the cutoffs, by risk doubling, into six groups with the bottom risk estimate <1% and subsequent cutoffs at 2, 4, 8, and 16%, respectively. How such a strategy would impact on clinical care and outcomes is speculative but might be interesting to investigate. Further, given that this is a single centre study, the contribution of each parameter would need to be established for different hospitals.

## 5. Conclusion

In conclusion, we have presented a nomogram for predicting 30-day in-hospital mortality for all medical admission patients admitted via the emergency department of our centre. The nomogram attempts to clarify and simplify risk prediction and offer an additional assistance to clinicians to understand how the relative contribution of different parameters overall impacts the hospital mortality risk of a patient. The nomogram offers a means of applying personalized decision analysis at the bedside or as an educational tool.

## Figures and Tables

**Table 1:** Logistic regression model to predict 30-day hospital mortality rate for the full model.

Predictor	Odds	Std. err.	*z*	*p* > *z*	[95% conf. interval]	
Acute Illness Severity	3.75	.16	30.4	0.001	3.44	4.08
Charlson Index	1.57	.04	16.5	0.001	1.49	1.66
Chronic Disabling Score	1.23	.03	9.9	0.001	1.18	1.29
Sepsis Term	2.14	.07	24.7	0.001	2.01	2.27
Manchester Triage	2.22	.08	22.2	0.001	2.07	2.39
Year effect	0.91	.004	−19.3	0.001	0.90	0.92
